# Intravenous contrast-enhanced CT can be used for CT-based attenuation correction in clinical ^111^In-octreotide SPECT/CT

**DOI:** 10.1186/s40658-015-0108-1

**Published:** 2015-02-12

**Authors:** Thomas Levin Klausen, Jann Mortensen, Robin de Nijs, Flemming Littrup Andersen, Liselotte Højgaard, Thomas Beyer, Søren Holm

**Affiliations:** Department of Clinical Physiology, Nuclear Medicine and PET, Copenhagen University Hospital Rigshospitalet, Blegdamsvej 9, DK-2100 Copenhagen, Denmark; Center for Medical Physics and Biomedical Engineering, General Hospital Vienna, Medical University of Vienna, Waehringer Guertel 18-20/4L, 1090 Vienna, Austria

**Keywords:** Combined SPECT/CT, Attenuation correction, CT contrast agents

## Abstract

**Background:**

CT-based attenuation correction (CT-AC) using contrast-enhancement CT impacts ^111^In-SPECT image quality and quantification. In this study we assessed and evaluated the effect.

**Methods:**

A phantom (5.15 L) was filled with an aqueous solution of In-111. Three SPECT/CT scans were performed: (A) no IV contrast, (B) with 100-mL IV contrast, and (C) with 200-mL IV contrast added. Scan protocol included a localization CT, a low-dose CT (LD), and a full-dose CT (FD). Phantom, LD and FD scan series were performed at 90, 120, and 140 kVp. Phantom data were evaluated looking at mean counts in a central volume.

Ten patients referred for ^111^In-octreotide scintigraphy were scanned according to our clinical ^111^In-SPECT/CT protocol including a topogram, a LD (140 kVp), and a FD (120 kVp). The FD/contrast-enhanced CT was acquired in both arterial (FDAP) and venous phase (FDVP) following a mono-phasic IV injection of 125-mL Optiray (4.5 mL/s). For patient data, we report image quality, Krenning scores, and mean/max values for liver and tumor regions.

**Results:**

Phantoms: in uncorrected emission data, mean counts (average ± SD) decreased with increasing IV concentration: (A) 119 ± 9, (B) 113 ± 8, and (C) 110 ± 9. For all attenuation correction (AC) scans, the mean values increased with increasing iodine concentration.

Patients: there were no visible artifacts in single photon emission computed tomography (SPECT) following CT-AC with contrast-enhanced CT. The average score of image quality was 4.1 ± 0.3, 3.8 ± 0.4, and 4.2 ± 0.4 for LD, arterial phase, and venous phase, respectively.

A total of 16 lesions were detected. The Krenning scores of 13/16 lesions were identical across all scan series. The max pixel values for the 16 lesions showed generally lower values for LD than for contrast-enhanced CT.

**Conclusions:**

In ^111^In-SPECT/CT imaging of phantoms and patients, the use of IV CT contrast did neither degrade the SPECT image quality nor affect the clinical Krenning score. Reconstructed counts in healthy liver tissues were unaffected, and there was a generally lower count value in lesions following CT-AC based on the LD non-enhanced images. Overall, for clinical interpretation, no separate low-dose CT is required for CT-AC in ^111^In-SPECT/CT.

## Background

Diagnostic imaging is essential in the workup of patients with a variety of diseases. Nuclear medicine imaging techniques, such as single photon emission computed tomography (SPECT) and positron emission tomography (PET), have for decades made an impact on the diagnostic pathways for a variety of indications in oncology, neurology, and cardiology [1–6]. Compared to PET, SPECT has the advantage of using radioactive labeled tracer molecules with relatively long physical half-lives that to a great extent are similar to the biological processes under observation. In general, isotopes for SPECT imaging are more easily attainable, and SPECT is generally more widely available than PET. Furthermore, SPECT has the ability, based on photon energy information, to perform dual isotope scans in a simple way. With recent advances in calibration, data processing, and, perhaps most importantly, the combination of SPECT and computed tomography (CT), SPECT has now become a quantitative, nuclear medicine imaging technique [7].

A major application of SPECT is in myocardial perfusion imaging [8,9]. This application and the wish to expand on its use have led to the promotion of separate transmission imaging as a pre-requisite to derive SPECT attenuation correction factors [10]. The increased wish for fast, noise free, and clinically viable SPECT attenuation correction (AC) was a primary drive for the development of combined SPECT/CT technology [11]. Therefore, the reasoning for the combination of SPECT and CT was distinctly different from that for the combination of PET and CT [12], which was developed to address a clinical need of fusing PET and CT images routinely and allowing for extended anatomical coverage adding only little additional scan time [13].

LaCroix and colleagues first presented concepts for CT-based attenuation correction (CT-AC) of SPECT data [14] long before the first presentation of a clinical SPECT/CT system [15]. Based on simulations with a mono-energetic X-ray beam, the authors showed that SPECT quantification of the myocardium was accurate to within 9% of the true activity concentration. Blankespoor and colleagues took on these studies and adopted CT transmission imaging and CT-based corrections for attenuation and scatter of SPECT in a prototype SPECT/CT system [16]. Further studies with their prototype SPECT/CT system and subsequent assessments of SPECT quantification in the heart and torso [17] have contributed to a great extent to the development of the first SPECT/CT system presented by GE Healthcare in 2000 [18]. Today, four major commercial vendors provide many different designs of SPECT/CT systems for clinical use, all of which provide CT-AC image reconstruction routinely.

With the dissemination of SPECT/CT, the range of applications has expanded from myocardial perfusion imaging to oncology indications, such as torso imaging in patients with neuroendocrine tumors (NETs), differentiated thyroid carcinomas, lymphoma, and sentinel node lymphoscintigraphy [2]. SPECT/CT imaging has also been shown to positively affect the localization of scintigraphic lesions, to aid in the differentiation of benign and malignant sites, and to guide biopsies, to name a few applications [19,20].

Several of these indications require the administration of CT contrast agents for enhancement of the vascular system and better differentiation of parenchymal tissue uptake. The use of CT contrast in combined imaging has been the subject of debate among PET/CT users [21,22], the reason being that standard, segmentation, and bi-linear scaling methods of CT-AC [23,24] fail to adequately scale contrast-enhanced tissues or filled organs, thus giving rise to artifacts and bias. However, most of these issues can be addressed by using modified imaging protocols [25].

Nonetheless, similar concerns among SPECT/CT users have limited the wider adoption of contrast-enhanced CT protocols as part of clinical SPECT/CT imaging. If CT contrast is indicated and used then most SPECT/CT imaging protocols entail a non-enhanced, low-dose CT for the purpose of attenuation correction and a contrast-enhanced CT for the purpose of advanced image fusion. Prior studies on the effect of CT contrast in SPECT/CT are sparse. Römer et al. reported [26] IV and positive oral contrast to have only a minor influence on calculated attenuation coefficients. Another study, using phantom data and IV contrast concentrations several times higher than those observed in clinical practice, has demonstrated a 36% overestimation of the AC-SPECT activity unit [27].

In this study, we assess the effect of IV contrast enhancement on ^111^In-SPECT image quality and quantification following CT-based attenuation correction using phantoms with clinically relevant iodine contrast concentration and in oncology patients.

## Methods

### Phantom study

**Figure 1:**
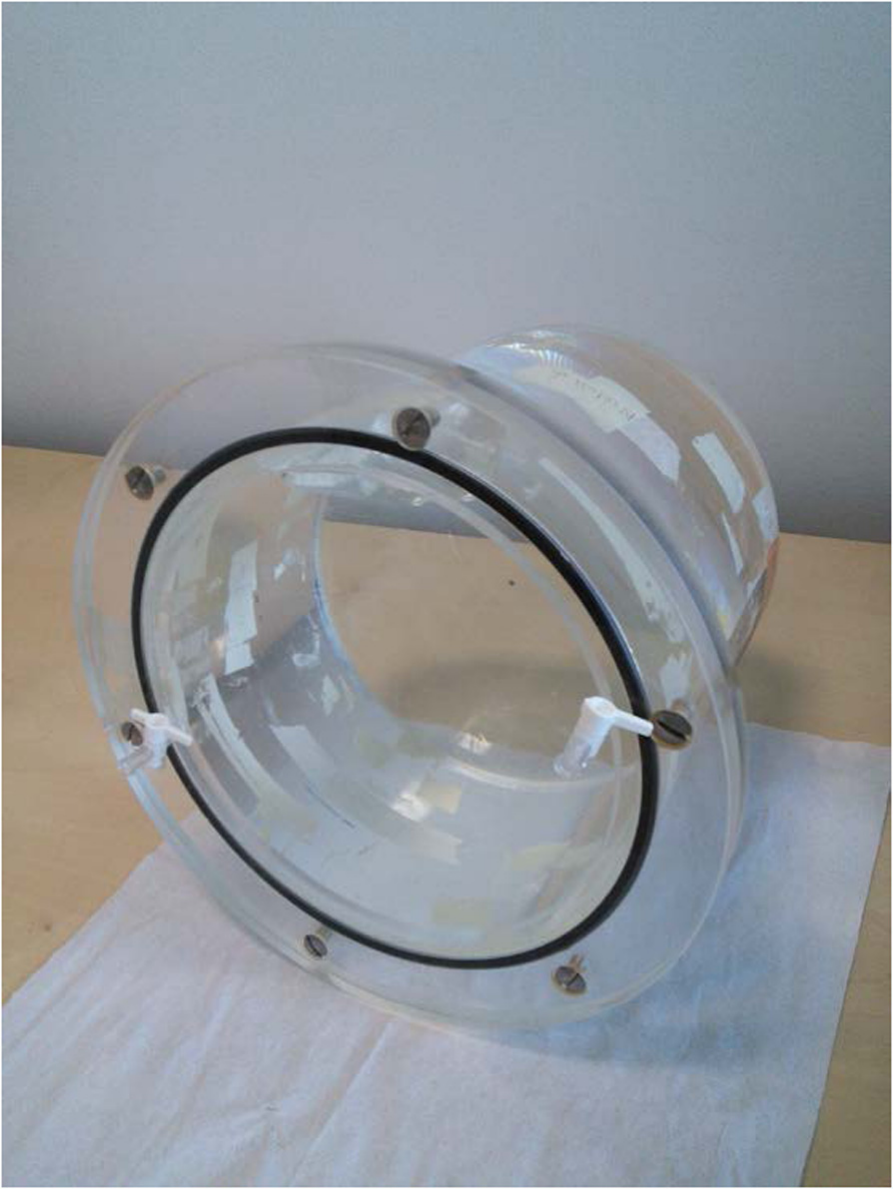
**A 20**-**cm cylinder phantom (5.15 L).**

A 20-cm cylinder phantom (5.15 L) was filled with water (Figure 1), and three separate imaging conditions were prepared for using IV contrast (Optiray, 350 mg/mL iodine, Mallinckrodt Pharmaceuticals, Hennef, Germany):

- (PC0) No IV contrast added
- (PC100) 100-mL IV contrast (equals 6.8 g iodine/L)
- (PC200) 200-mL IV contrast (equals 13.6 g iodine/L)

For each of the three phantom conditions (PC0, PC100, PC200), 50 MBq In-111 was added to the water solution in the cylinder (10 kBq/mL). Based on our clinical protocol, 50 MBq was chosen as a reasonable number with an injection of 200 MBq 48 h before scan. The active phantom was centered in the field-of-view of a clinical SPECT/CT system (Philips Precedence 16 slice CT, two-headed gamma camera, Philips Medical Systems, Eindhoven, the Netherlands) for imaging. For each of the phantom conditions, the SPECT/CT protocol included a localization scan, spiral CT, and a single-bed SPECT acquisition (128 projections, 20 s/position, matrix size 128 × 128, scan time 22 min) using two energy windows centered at 171 and 245 keV with a width of 20% and a medium energy general purpose (MEGP) collimator. In each protocol, the spiral CT was acquired as:

- a low-dose CT (LD): 38 mA, 5-mm slice thickness, pitch 0.94, rotation time 0.5 s and
- a full-dose CT (FD): 469 mA, 2-mm slice thickness, pitch 0.94, rotation time 0.5 s

**Figure 2:**
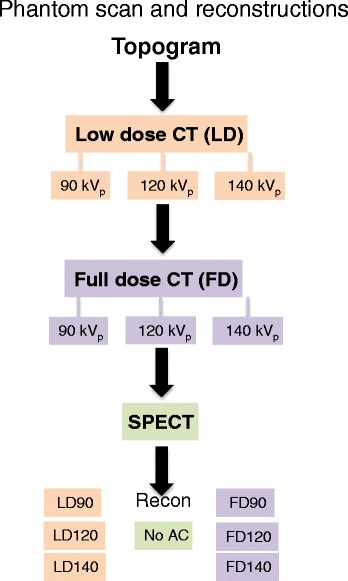
**Overview of scan flow and reconstructions.** A total of seven SPECT data sets were reconstructed for each scan series; one noAC and three AC based on LD with different kVp and three AC based on FD with different kVp.

In both LD and FD scan series, the CT scans were performed at 90, 120, and 140 kVp yielding for each phantom preparation that were used a total of six different datasets as input to AC-SPECT image reconstruction. The μ-maps for AC were derived for an energy corresponding to an (weighted) average of the two energy windows using standard vendor software. In addition, the SPECT images of all three phantom conditions were reconstructed without AC (noAC). In total, the three phantom sets each comprise one noAC-SPECT and six AC-SPECT images. All SPECT data were reconstructed iteratively using the Philips Astonish algorithm including the built-in scatter correction but without additional filtering: 4 iter/16 sub, pixel size 4.66 mm × 4.66 mm. Figure 2 summarizes the reconstruction pathways and reconstructed image data.

### Patient study

Ten patients (seven males, three females) referred for ^111^In-octreotide scintigraphy were consecutively enrolled in the study (from August 2012). All patients had confirmed SPECT-positive lesions.

The mean age was 63.4 years (range 53 to 76 years) and the mean weight was 75 ± 19 kg with an average BMI of 23.7 ± 2.5. Patients were injected with 220 ± 16 MBq ^111^In-pentetreotide (Octreoscan™) from Mallinckrodt Pharmaceuticals, Hennef, Germany, and scanned 48 h post-injection. All patients were positioned in the head-first supine position with arms up. The relevant areas for the combined SPECT/CT examination were defined based on a prior whole-body planar scintigraphy. The axial field-of-view typically covered the abdomen or the thorax, depending on the findings from the scintigraphy scan.

All patients were scanned according to our clinical ^111^In-SPECT/CT protocol including a topogram, a LD (140 kVp, 38 mA, 5-mm slice thickness, pitch 0.94, rotation time 0.5 s), a FD (120 kVp, 469 mA, 2-mm slice thickness, pitch 0.94, rotation time 0.5 s), and a single-bed SPECT scan (using two energy windows centered at 171 and 245 keV with a width of 20% ,MEGP collimator, 128 projections, 20 s/position, matrix size 128 × 128, scan time 22 min). The FD contrast-enhanced CT was acquired in both the arterial and venous phase following a mono-phasic IV injection of 125 mL Optiray (4.5 mL/s). The time delay between arterial phase and venous phase was 70 s. CT images were reconstructed into 512 by 512 matrices.

SPECT images were reconstructed without (noAC) and with AC. Iterative SPECT image reconstruction was performed using the implemented Astonish algorithm with scatter correction and without additional filtering: 4 iter/16 sub, pixel size 4.66 mm × 4.66 mm. AC-SPECT data were reconstructed with the three CT series: LD, FD in the arterial phase (FDAP), and FD in the venous phase (FDVP).

During the initial reconstruction steps, an experienced nuclear medicine physician visually inspected the CT and noAC-SPECT images for misalignment. If a noticeable misalignment was detected, the CT image volume was realigned manually to match the noAC-SPECT and CT-AC was performed followed by image recon (Table 1).

**Table 1:** **Visually based misalignment (values in mm) between LD, FDAP, and FDVP and their corresponding SPECT data**

**Patient #**	**LD_MC**				**FDAP_MC**				**FDVP_MC**			
	**x**	**y**	**z**	**Rotation**	**x**	**y**	**z**	**Rotation**	**x**	**y**	**z**	**Rotation**
1	0	0	−10	0	0	0	−12	0	0	0	−28	0
2	0	0	0	0	0	0	0	0	0	0	−18	0
3	0	0	0	0	0	−15	−31	0	0	−10	−30	0
4	0	0	0	0	0	−5	−20	0	0	−5	−30	0
5	0	0	0	0	0	−10	−30	0	0	−10	−30	0
6	0	0	0	0	0	−10	−20	0	0	−10	−20	0
7	0	0	0	0	0	−10	−25	0	0	−10	−25	0
8	0	0	0	0	0	0	0	0	0	0	−15	0
9	0	0	0	0	0	−5	0	0	0	−5	0	0
10	0	0	0	0	0	0	0	0	0	0	0	0

### Data evaluation

#### Phantoms

For all scan series, we report the average volume of interest (VOI) value (reconstructed counts) calculated from central circles with a radius of 5 cm placed on 11 central image planes of the phantom.

#### Patients

An experienced nuclear medicine physician with over 10 years of clinical SPECT experience performed clinical evaluation of all AC-SPECT images. All reconstructions were evaluated blinded and presented in random order. On this data the following were performed:

- SPECT images were screened for image artifacts (yes/no) and assessed for overall image quality using a scale of 5 (grade 1 = very poor, 2 = poor, 3 = acceptable, 4 = good, 5 = very good).
- SPECT-positive lesions were detected and values (reconstructed counts) for the hottest lesion in each organ were reported.
- The lesions were scored visually using the 4-point Krenning score scale [28]; lower than (grade 1), equal to (grade 2), or greater than (grade 3) normal liver tissue; or higher than normal spleen or kidney uptake (grade 4).

Fourth, lesion max values were reported, and fifth, we report the average value of a 10-cm^2^ circular region of interest (ROI) placed on healthy liver tissue in a region with no or little IV contrast uptake.

### Statistical analysis

A paired t-test (two-tailed) was used for comparison of reconstructed counts in the lesions and the liver between the LD, FDAP, and FDVP reconstructions. The significance level was set to *p* < 0.05.

## Results

### Phantom studies

**Figure 3:**
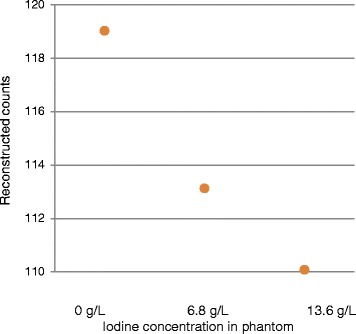
**noAC In-111 reconstructed counts for each of the three phantom conditions.** noAC In-111 reconstructed counts for each of the three phantom conditions PC0, PC100, and PC200 corresponding to 0, 6.8, and 13.6 g/L iodine in phantom, respectively.

**Figure 4:**
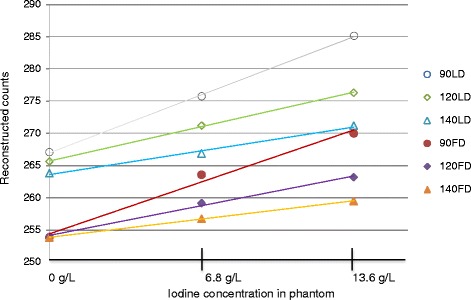
**SPECT mean reconstructed count value as a function of increasing iodine concentration.** All data are AC using LD at 90, 120, and 140 kVp or FD at 90, 120, and 140 kVp.

With uncorrected emission data (noAC), mean counts decreased with increasing iodine concentration (Figure 3). For all AC scans, the mean reconstructed count value increased as a function of increasing iodine concentration. The slope of this function decreased with increasing kVp, and the slope values were higher for LD than for FD (Figure 4, Table 2). Furthermore, for all AC scans, the mean counts for LD were about 5% higher than for the corresponding FD.

**Table 2:** **The** **slope of the mean values (reconstructed counts) as a function of increasing iodine concentration**

**Tube voltage (kVp)**	**Slope LD (mean counts (g/L) iodine)**	**Slope FD (mean counts (g/L) iodine)**
90	1.32	1.19
120	0.79	0.68
140	0.54	0.42

The slope decreased with increasing kVp, and the values were higher for LD than for FD.

### Patient studies

**Figure 5:**
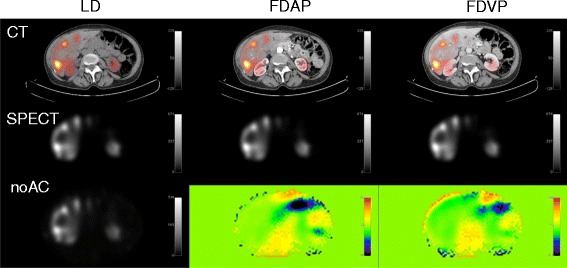
**CT images.** Top row: CT images for LD, FDAP, and FDVP. Middle row: AC-SPECT images for LD, FDAP, and FDVP. Lower row: first image noAC-SPECT images; second images percent difference between LD- and FDAP-SPECT; third image percent difference between LD-FDVP-SPECT.

There were no visible artifacts in the SPECT images following CT-AC with the LD, FDAP, or the FDVP (Figure 5).

The average score of SPECT image quality was 4.1 ± 0.3, 3.8 ± 0.4, and 4.2 ± 0.4 for LD, FDAP, and FDVP, respectively. The t-test showed a significant difference (*p* < 0.04) between FDAP and FDVP only (Table 3). In total, 16 SPECT-positive lesions were evaluated with max pixel value of 293 ± 214, 312 ± 226, and 307 ± 218 for LD, FDAP, and FDVP, respectively (Table 3).

**Table 3:** **Image** **score quality for the 10 patients showed significant difference (** ***p*** **< 0.04) between FDAP and FDVP only**

**Patient #**	**Image score**			**Lesion value (max pixel)**			**Krenning score**	**Lesion location**
	**LD**	**FDAP**	**FDVP**	**LD**	**FDAP**	**FDVP**	**LD, FDAP, FDVP**	
1	4	4	5	498	516	446	4, 4, 4	Liver
				135	145	146	3, 3, 3	Lymph Node
2	4	3	4	678	671	661	4, 4, 4	Liver
3	4	3	4	49	46	55	3, 3, 3	Liver
				158	171	174	3, 3, 3	Lymph Node
4	4	4	4	753	813	790	4, 4, 4	Lymph Node
5	4	4	4	270	320	309	3, 3, 3	Pancreas
6	5	4	4	142	162	168	3, 3, 3	Lymph Node
7	4	4	5	65	67	68	3, 3, 3	Liver
				543	593	593	4, 4, 4	Lymph Node
8	4	4	4	249	295	290	3, 4, 4	Liver
				186	186	192	3, 3, 4	Bone
9	4	4	4	131	133	130	3, 3, 3	Liver
				358	385	385	3, 4, 4	Lymph Node
10	4	4	4	382	410	423	4, 4, 4	Liver
				89	83	81	3, 3, 3	Lymph Node
Average	4.1 ± 0.3	3.8 ± 0.4	4.2 ± 0.4	293 ± 214	312 ± 226	307 ± 218	4.1, 3.8, 4.2	N/A

Max pixel values for the 16 lesions shows significantly lower values for LD than for FD; *p* < 0.003 and *p* < 0.05 for arterial and venous phase, respectively. On the average, LD values are 6% ± 7% and 6% ± 8% lower. For 13 out of 16 lesions, the Krenning score is identical in all the CT phases. In the remaining three lesions (highlighted), the Krenning score differed by no more than one.

For 13 out of 16 lesions, the Krenning score was identical in all the CT phases. In the remaining three lesions, the Krenning score differed by no more than one (Table 3).

Comparison of max pixel values for the 16 lesions showed significantly lower values for LD than for FD; *p* < 0.003 and *p* < 0.05 for arterial and venous phase, respectively. On average, LD values were 5.8% ± 7% and 6.1% ± 8% lower.

Across all 10 patients, there were no significant differences in the mean count value for the liver ROI (Table 4). However, in individual patients, there were differences up to around 8%. The highest difference between the three scans series was between the two FD series with FDVP (venous phase) on average being 2.1% ± 3% higher than FDAP (arterial phase).

**Table 4:** **Reconstructed counts from liver ROI**

	**Reconstructed counts from liver ROI**		
**Patient #**	**LD**	**FDAP**	**FDVP**
1	78.4	80.3	80.3
2	84.2	84.7	90.2
3	22.4	21.0	22.6
4	56.1	54.0	54.9
5	49.3	48.8	49.8
6	45.5	44.2	44.8
7	26.3	25.1	25.3
8	22.7	22.3	22.9
9	44.6	47.2	47.1
10	33.6	33.0	32.3
	t-test (p-values) 0.58	0.34	0.11

Mean reconstructed count values from a 10 cm^2^ ROI (circle) placed on healthy liver tissue in a region with little IV contrast uptake.

## Discussion

Our study demonstrates that a CT scan with an iodine-based IV contrast agent can be used for CT-based attenuation correction of a ^111^In-SPECT scan in combined modality with SPECT/CT scanning. No artifacts were seen in the SPECT images, and the clinical interpretation using Krenning score did not change significantly. However, changes in quantitative values in the range of 6% were observed. So for clinical interpretation without high-quantitative accuracy, no separate low-dose CT is required for CT-AC in ^111^In-SPECT/CT.

Administration of iodine-based contrast increases the attenuation by increasing the photoelectric absorption of photons in the energy interval of interest in CT and in SPECT, and this effect is evident in the uncorrected SPECT data (Figure 3). However, due to the low-mean energy of the Bremsstrahlung spectrum from the CT compared to most radioisotopes used in SPECT, and the rapid decline of photoabsorption with energy, the attenuation enhancement is higher in the CT acquisition than in the SPECT acquisition. When CT is used for attenuation correction, a conversion of Hounsfield Unit (HU) into attenuation values at the proper SPECT energy is performed [14]. This conversion assumes a certain tissue composition (dependent on the HU) and does not account for the presence of iodine. The result will be an overestimation in the attenuation values used for the correction of the emission data and, thus, results in a higher activity concentration in the reconstructed emission image. The dependency on CT energy, as seen by the decreasing slope with increasing CT energy (Table 2), is consistent with this.

The shift in average attenuation values may propagate through CT-AC and cause local artifacts that may be incorrectly interpreted in the clinical reading. This was discussed by Roemer et al. [26] when presenting findings where the presence of CT contrast had a clinically relevant effect on SPECT quantification.

Their findings are in line with this study that indicate generally lower count values (6%) in ^111^In-SPECT-positive lesions following CT-AC using LD compared to contrast enhanced CT (Table 3).

To obtain quantitative images in PET and SPECT, many corrections such as attenuation, scatter, and partial volume correction need to be taken into account during image reconstruction. The AC challenges for SPECT are larger by far compared to PET, in essence, due to the fundamental physical differences in decay scheme for PET and SPECT isotopes and the subsequent differences in scanner design [30]. However, by applying attenuation, scatter, and partial volume correction, activity estimations can be obtained with error levels of 3% to 5% for Tc-99m, In-111, I-123, and I-131, as shown by Shcherbinin et al. [29] in phantom studies. In clinical settings, SPECT with Tc-99m can be quantitative with errors less than 5% to 10% [30–32]. With errors on the order of just 10%, quantitative SPECT can be promoted further in a clinical setting [7]. On the scanner side, vendors are also starting to move towards quantitative SPECT/CT [33], but so far, only quantitative SPECT for Tc-99m has been implemented.

As mentioned above, CT attenuation correction based on contrast-enhanced CT affects the SPECT quantification; nevertheless, the effect is small and similar to the one seen in PET. Therefore, it is likely that the continuing development/use of quantitative SPECT in the clinic can include IV-contrast-enhanced CT and still maintain the overall goal to provide reliable and accurate images.

This is relevant because, as shown by Wieder et al. [34], SPECT/CT is not used to its full potential, mainly because SPECT/CT is operated in nuclear medicine departments only, but also because contrast is rarely ever used; this study shows that the additional LD can be spared and IV contrast can be used. This will help reduce patient exposure and scan time, and using IV contrast in combined modality with SPECT/CT will be more cost effective since the patient will be spared a standalone CT scan. Furthermore, as it is the case for PET/CT, a SPECT/CT scan with a diagnostic quality CT including IV (and oral) contrast agents is advantageous compared with a CT scan performed with IV contrast on one occasion plus a SPECT/CT with LD for image fusion and attenuation correction performed on another occasion. To our knowledge, the latest guidelines from the European Association of Nuclear Medicine (EANM) or the Society of Nuclear Medicine and Molecular Imaging (SNMMI) dates back to 2006 [35] and should be adhered to regarding the potential increasing use of CT in combination with SPECT.

The number of patients included in this study was small, and the anatomical distribution of lesions shows little variation (14 out of 16 lesions were located in the liver or lymph nodes, Table 3). A bias due to patient selection is a possibility, but there is no obvious reason to believe that a different lesion distribution should be more sensitive to the presence of IV contrast. However, one could consider demonstrating the effect of IV contrast in a larger patient group with a different pathology.

Differences in breathing status in a combined modality scanner are among the most prominent problems for the use of CT data in attenuation correction. The misalignment adds bias to SPECT images [36], and therefore, at clinical evaluation of attenuation-corrected scans, special attention should be given to possible misalignments between SPECT and CT. If there is a misalignment, ideally, a manual realignment of CT and SPECT images should be performed followed by a new reconstruction. In the present study, misalignments between SPECT and CT were seen in 9 out of 10 patients.

## Conclusions

In ^111^In-SPECT/CT imaging of phantoms and patients, the use of IV CT contrast did neither degrade the SPECT image quality nor affect the clinical Krenning score. Reconstructed counts in healthy liver tissues were unaffected, and there was a generally lower count value in lesions following CT-AC based on the LD non-enhanced images. Overall, for clinical interpretation, no separate low-dose CT is required for CT-AC in ^111^In-SPECT/CT.
